# Direct Observation of Dog Density and Composition during Street Counts as a Resource Efficient Method of Measuring Variation in Roaming Dog Populations over Time and between Locations

**DOI:** 10.3390/ani7080057

**Published:** 2017-08-03

**Authors:** Elly Hiby, Lex Hiby

**Affiliations:** Conservation Research Ltd., 110 Hinton Way, Great Shelford, Cambridge CB22 5AL, UK; lexhiby@gmail.com

**Keywords:** dog, stray dog, dog population management, animal welfare, survey, population density, strip transect, monitoring, evaluation

## Abstract

**Simple Summary:**

Roaming dogs are a common sight in many countries; they can be undernourished and unwell and may be a risk to public health. Different ways of improving the situation are proposed and attempted in many locations. To see which work, we suggest measuring dog density by counting dogs along standard routes across locations and repeating those counts at the same time of year and day, being careful to count the same way each time. It is not necessary to estimate the total number of dogs because it is the number per km of street that determines how many dogs a resident will meet on their way to school or work, and reducing density and healthier dogs would be considered a success. Smartphone applications make it easy to stick to standard routes and record dogs of different types, so we can also track things like the percentage of females with pups and the percentage of dogs that are emaciated. We present examples of such counts demonstrating large differences in densities between countries. There are few examples of counts over several years but we present one showing a definite reduction in density in a location that has provided spay and neuter services for several years.

**Abstract:**

Dog population management is conducted in many countries to address the public health risks from roaming dogs and threats to their welfare. To assess its effectiveness, we need to monitor indicators from both the human and dog populations that are quick and easy to collect, precise and meaningful to intervention managers, donors and local citizens. We propose that the most appropriate indicators from the roaming dog population are population density and composition, based on counting dogs along standard routes using a standard survey protocol. Smart phone apps are used to navigate and record dogs along standard routes. Density expressed as dogs seen per km predicts the number of dogs residents will encounter as they commute to work or school and is therefore more meaningful than total population size. Composition in terms of gender, age and reproductive activity is measured alongside welfare, in terms of body and skin condition. The implementation of this method in seven locations reveals significant difference in roaming dog density between locations and reduction in density within one location subject to intervention. This method provides a resource efficient and reliable measure of roaming dog density, composition and welfare for the assessment of intervention impact.

## 1. Introduction

Domestic dogs have evolved alongside humans for many thousands of years, and are currently almost ubiquitous in human society. Global estimates of dog population size are over 700 million [1]. In many countries, owned dogs are allowed to roam freely outside their owner’s property for at least a portion of the day. For example, 68.5% of dogs were reported by their owners to be unconfined for at least part of the day in Todos Santos, Guatemala [2]; this was 37% in Iringa, Tanzania [3]; and between 27% and 67% in different sized urban areas in the Coquimbo region of Chile [4]. In addition, those dogs that do not have an owner also roam freely in the same space, in search of resources such as shelter and food, in the form of edible garbage and handouts from sympathetic people. The presence of roaming dogs, both owned roaming and unowned dogs, on streets and public property is therefore not uncommon. The density of this roaming dog population appears to differ between locations; however, there is limited objective data exploring this variation.

Due to the public health risks that these dogs may present and the threats to their own welfare many governments, municipalities and non-governmental organisations implement interventions to manage dog populations. The goals are often to reduce the number of dogs on the street and to improve the welfare and safety of the remaining population. However, there is limited published data detailing the impact of these efforts on dog density or welfare. In a scoping review of the literature [5], 26 studies assessing the impact of dog population management (DPM) were found, including seven which attempted to measure the impact on dog population density, of which only two were in peer-review journals [6,7], the remaining four were a book, conference proceedings or unpublished reports. This paucity of data leaves those responsible for managing dog populations limited in their ability to make evidence-based decisions on which interventions to establish, or to evaluate and improve the impact of their own interventions. Further, a failure to present evidence of impact may reduce the chances of ongoing funding and the political will to sustain an intervention.

An estimate of the total roaming dog population may be requested for planning or evaluating an intervention. However, this measure of abundance is time-consuming to establish, requiring repeated surveys of naturally or artificially marked samples of roaming dogs (for example [7] and [8]); the estimates are also subject to biases that are difficult to quantify. Abundance also requires clear definition in the context of a population consisting of a mixture of unowned and unconfined owned dogs, should it for example include those owned dogs that may only venture out of their owner’s property for a few minutes each morning? Or should some threshold of roaming apply, such as only those owned dogs that roam for at least 20% of the day? Such definitions rely upon the knowledge of owners, which is time-consuming to measure and will be subject to unquantifiable error. Where the budget available for research is generous, assessing well defined dog population abundance, including sub-populations of dogs with different levels of ownership and confinement, may be possible; however, this is rarely the case in those locations where roaming dog density is a significant problem. An alternative is to focus on roaming dog population density as the appropriate indicator for evaluating the impact of a DPM intervention. We can define density as the expected number of roaming dogs (regardless of ownership status which is usually unidentifiable at the time of observation), that would be encountered by trained observers moving along every street within the region of interest divided by the total surveyed street length. Again, we need additional specifications: how fast observers should move, at what time of day and year, the extent to which observers should search under cars and so on. It is however easy to comply with such specifications once defined.

Assuming that there is insufficient time to move along every street, we can estimate population density by selecting a random sample of streets, counting the roaming dogs encountered along that route and dividing by its length. To select a purely random sample, each unit of street length within the region of interest would need to have the same probability of inclusion. Although we cannot ensure that is the case, we can try to make the selected route representative of the region of interest by having the route drawn by someone naïve to the expected spatial variation in roaming dog density. Any resulting biases affect comparison of density between different regions, but by using the same route for successive surveys we avoid bias invalidating estimates of change over time on that route.

A notable benefit of this measure is that most citizens are not affected directly by the total number of roaming dogs in their city whereas roaming dog density, expressed as dogs seen per km, determines the number of dogs they will encounter as they commute to a regular destination, for example their place of work or school. Hence, a change in roaming dog density along public streets is more meaningful to the potential beneficiaries of the intervention. In addition, most urban areas are growing in size; in the developing world this rate of growth can be staggeringly high. In such an urban area, the total number of roaming dogs may increase along with expansion and development of the city. Thus, even if an intervention succeeds in reducing the density of roaming dogs on a city’s streets to the benefit of the residents, it may be incorrectly deemed to have failed if the absolute population size has not reduced. Finally, if carefully conducted, measurements of population density have much higher precision than estimates of total abundance obtained using the same level of effort, and are therefore more sensitive to any changes resulting from intervention.

Observers also have the opportunity to record the type of each dog encountered and its visible body and skin condition, resulting in estimates of the composition of the roaming dog population. Interventions commonly include reproduction control to reduce unwanted puppies and breeding behaviours that are considered a nuisance. By monitoring the percentage of females that show visible signs of lactation, the extent to which the intervention is accessing the roaming dog population can be assessed. In addition, monitoring body and skin condition may provide a measure of an intervention’s impact on dog welfare.

The authors hence set out to establish a method of measuring roaming dog population density and composition that would be sensitive to differences between location and changes within location over time, but also simple and cost effective to implement. It is an adaptation of index monitoring, primarily used for wildlife (for example, see [9] and [10] for discussions of ideal index monitoring methodologies and analyses in several wildlife species), to a domestic species. It assumes constant average detectability of roaming dogs by applying consistent search effort over time, achieved by observers adhering closely to the survey protocol and routes. The methodology utilises counts of roaming dogs along routes following urban and suburban streets, effectively providing strip transects along the roads, pavements and verges between buildings to measure density [11]. Utilising the physical structures of buildings to create strip transects has been used for measuring density of other species in urban areas, including bird species, e.g., [12]. The method presented here has been trialed in several locations and refined based on implementation experience and results. It builds on the method utilised by Reece and Chawla [6] in Jaipur, India, where counts of roaming dogs are conducted along streets with almost exhaustive coverage of a particular area of the city. However, the method described here utilises routes that extend across urban areas to integrate over spatial variation in roaming dog density.

## 2. Materials and Methods

**Figure 1:**
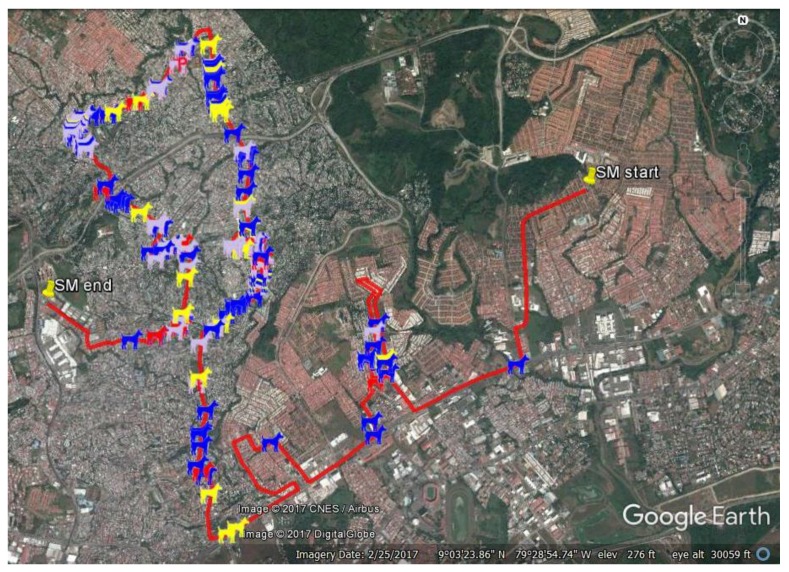
Google Earth image of the “San Miguelito” route in Panama City. The red line shows the route surveyed, the dog icons show the location of the observer when the roaming dog was recorded; blue dogs are male, yellow dogs are female, grey dogs are adults of unknown gender and red dogs are lactating females. Where a sterilisation programme has taken place, using visible marks of sterilisation status such as ear notches or ear tags, black and green icons are used to represent castrated males and spayed females.

To measure change in population density we need a standard unit of survey effort (e.g., “catch per unit effort” in fisheries). In the case of roaming dogs we selected the number of roaming dogs seen per kilometre along standard routes using a standard survey protocol. As these same routes must be maintained for all future surveying the initial selection of routes must be done with care. Routes were drawn using the “add driving route” tool in Google My Maps (www.google.co.uk/maps/about/mymaps); two approaches to designing routes were used:

- Representative routes. A “representative” route is drawn by someone naïve to the expected spatial variation in roaming dog density. The route is drawn to take in an approximately representative proportion of residential and larger roads, and areas of different housing types using the satellite view in My Maps (see Figure 1).
- “Hotspot” routes. The term “hotspot” was used because these routes were drawn to focus on areas known to have high density of roaming dogs. People local to the area with an interest in roaming dogs were asked to indicate on maps where they tended to see the most roaming dogs. The routes were then drawn to bisect as many of these areas as possible within the shortest distance.

Where representative routes are used, the average number of dogs per km of street can be extrapolated to make an estimate of the total number of dogs visibly roaming on public streets at the time of the survey, using the total road length for the area. Where an estimate of detectability is available, this can be used in a further stage of extrapolation to estimate the total number of roaming dogs at the outset of an intervention.

Navigation along the routes was supported by the use of mapping apps on smart phones. The routes were saved as kml files and then displayed on mapping apps, such as Google Maps, Maps.Me or Locus.

Direct observation of the dogs was done by a team of at least two observers. These observers would walk, cycle, drive or ride a motorcycle along the route. The mode of transport was chosen according to the accessibility of the roads and the preference of the surveyors. Once a mode of transport was selected this had to be maintained for future monitoring surveys.

The length of the route was dictated by the mode of transport as the aim was to create a route that took no longer than 2 h to complete, usually 20 km maximum in length when using a car or motorcycle.

Observers move as quickly as possible along the route, allowing sufficient time to assess gender and welfare, but attempting to move faster along the route than the dogs to avoid double counting. Interaction with the dogs is minimised to avoid influencing their movement; this either encourages them to come with observers or causes them to run away which increases the chances they will interact with other dogs. Interactions between dogs may lead to increased movement, therefore greater potential for double counting and risks aggressive encounters which endanger dog welfare and human safety.

A survey event required each route to be surveyed at least twice to establish a measure of day-to-day variation that could then be used to calculate the significance of any observed change in dog density over time.

**Figure 2:**
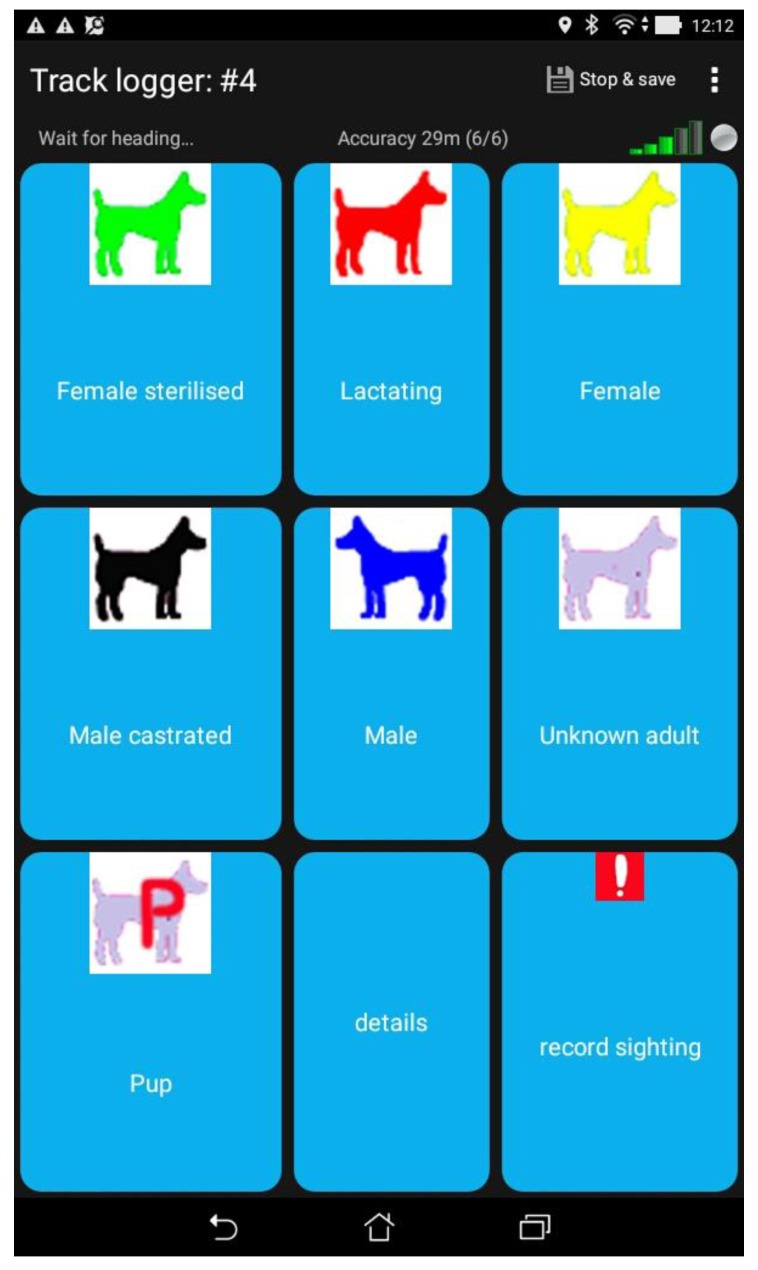
Image of OSMtracker app display on a mobile phone, showing an icon for each dog type recorded during surveying.

Observation of dogs was recorded using the smart phone app OSMtracker with a layout designed specifically for dog surveys; the observer taps an icon on the app display to record the observed dog type (see Figure 2). This is an event recording app that notes the GPS location of the observer at the time of event logged (see Figure 1 for an example of dog type icons displayed on Google Earth using GPS information recorded at the time of observation). Each dog was scored for the following:

- Gender/age
  ○ Male
  ○ Female
  ○ Lactating female
  ○ Unknown adult
  ○ Pup (under 4 months of age)
  ○ Sterilised male (only in those locations where marking of sterilised dogs is used)
  ○ Sterilised female (only in those locations where marking of sterilised dogs is used)
- Visible welfare
  ○ Body condition score (BCS) on a 5 point scale (using visual cues only, as described in [13])
  ○ Presence or absence of a visible skin condition

In each location, the protocol used for survey was recorded to ensure future monitoring surveys were conducted with consistent search effort. This included the start time for each route, average speed, mode of transport, acceptable weather conditions (e.g., do not survey during rain as fewer dogs will be visible), whether the observers should check under parked cars and how far down a side street off their route should they look.

Data were uploaded to an Access database and analysed. The differences between location in percentage of females lactating, the percentage of dogs with low body condition and the percentage with skin conditions were tested using a Kruskal-Wallis *H*-test. The difference in dogs per km between locations was tested using Analysis of Variance (ANOVA), differences in dogs per km over time and lactation over time within Panama City were tested using regression analysis.

### 2.1. Power to Detect Change in Density

We consider the situation where a survey following some level of intervention is to be compared to a baseline survey conducted prior to the intervention to detect any significant change in average density. Replicating the baseline surveys along each route then allows the power to detect change to be estimated simply as a minimum required observed change. The differences between the densities recorded on the replicate surveys, averaged over the routes and then divided by their standard error has a Student’s *t* distribution based on *n*−1 degrees of freedom where *n* equals the number of routes. Those differences can therefore be used to calculate the minimum percentage change observed by a future survey that would be required to detect a significant change in actual population density.

For example, a roaming dog survey of Mumbai in January 2014 [14] counted dogs seen per km along 21 predesigned routes, repeating the counts on the following day. A mean of 10.54 dogs were seen per km. As expected, the average difference between the replicate counts was close to zero at 0.097, with a standard error of 0.29. To use those same routes to show a change in current roaming dog density as compared to January 2014, significant at the 95% level, would therefore require that currently observed density is less than or more than 10.54 times 0.29 times *t*_0.05,20_, i.e., by approximately 6% or 0.63 dogs per km.

If a series of surveys are conducted, then selecting a pair that happen to show a desired change would invalidate the size of the test, which would need to be based on just the final survey or on a time series analysis and therefore a model of change over time.

### 2.2. Power to Detect Change in Composition

Replicate counts of roaming dogs of certain types, for example of lactating females, can also be used to estimate the power to detect change in their population density. However, the power to detect change in, for example, the percentage of females that are lactating can be estimated without replication if the number of lactating females counted can be assumed to have a binomial distribution. In that case the fraction of females that are seen to be lactating has approximately a normal distribution with standard deviation $\sqrt{\frac{1}{n}p(1-p)}$ where *n* equals the number of females counted and *p* equals the fraction that were lactating. Under a null hypothesis that the current population fraction lactating is unchanged as compared to an earlier survey, the difference between current and previous observed fractions would have zero expectation and standard deviation $\sqrt{(\frac{1}{n_{1}}+\frac{1}{n_{2}})p\left(1-p\right)}$ where *p* is a combined estimate based on the *n*_1_ and *n*_2_ females counted in the two surveys. The observed fractions would then have to differ by 1.96 times that estimated standard deviation to reject the null hypothesis and conclude at the 95% level that a change in the fraction lactating had occurred.

Using the Mumbai survey as an example; 0.08 of the 3236 females recorded during the street counts were lactating whereas 0.11 of the females counted in “slum” areas were lactating, an increase of 0.03. However the total number of females counted in the slum areas (*n*_2_) was less than the 463 required to make the increase in the observed fraction significant at the 95% level.

## 3. Results

**Figure 3:**
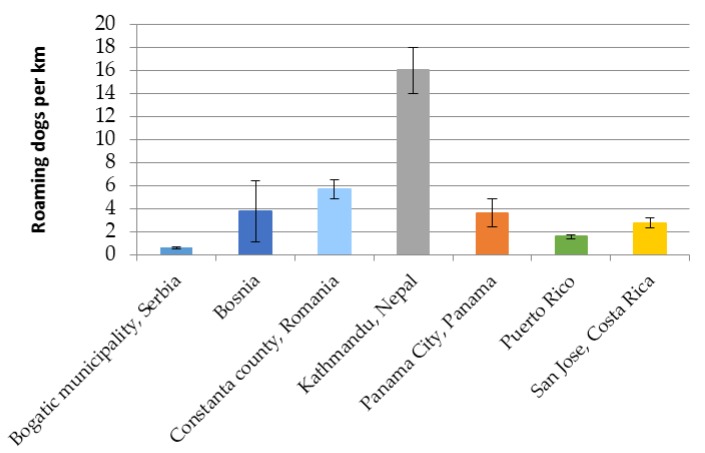
Bar chart showing the difference in average roaming dog density per km along both hotspot and representative routes in seven locations (it should be noted that both the design of the routes and the degree of urbanization along these routes varies between location, hence the average density along routes cannot be taken to represent national or regional roaming dog density), error bars shown are +/− one standard error.

This method has been conducted in many locations to date. Table 1 shows the data from 7 of these locations across Europe, Latin America and Asia. The number of roaming dogs observed per km of street surveyed in these locations is also shown in Figure 3.

The density of roaming dogs per km differed significantly between the locations listed in Table 1 (*F* = 10.943, *df* = 6, *p* < 0.001). Table 1 also provides three additional indicators. The percentage of females that are lactating, which did not differ significantly between country (*H* = 3.450, *df* = 6, *p* = 0.751); and two indicators of welfare state, visible skin problems and body condition score. The percentage of dogs with a visible skin problem showed a significant difference between the locations (*H* = 20.621, *df* = 6, *p* < 0.01). Body condition score was explored through both the percentage of dogs that were recorded as BCS 1 emaciated and the percentage that were either BCS 1 emaciated or BCS 2 thin. Neither of these indicators were significantly different between locations (BCS 1 *H* = 10.690, *df* = 6, *p* = 0.098; BCS 1 or 2 *H* = 8.907, *df* = 5, *p* = 0.179).

Monitoring using the same survey protocol has been carried out in some of the locations listed in Table 1. Table 2 displays the monitoring data from five locations, the number of repeat surveys that have been conducted ranging from 1 to 4.

**Figure 4:**
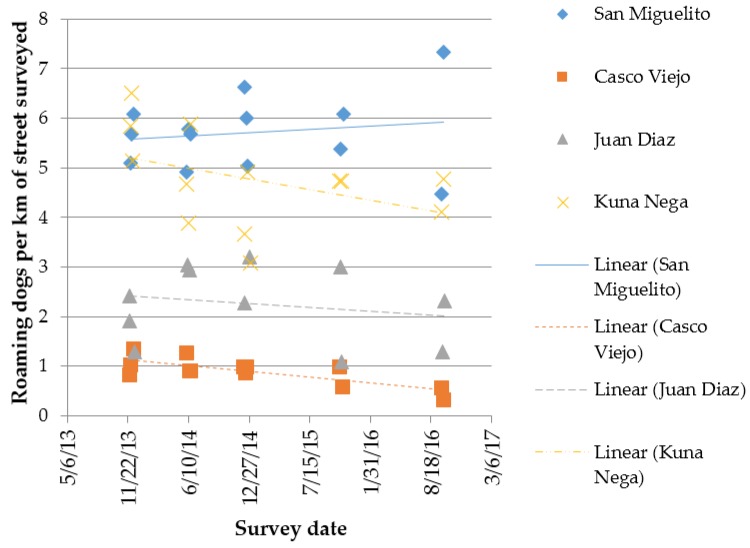
Roaming dogs per km of street surveyed for all four Panama City routes, showing the data for the two or three replicates at each survey event and five survey events across time.

The dogs per km of street survey in Table 1 and Table 2 are averages within route, resulting from two or more replicate surveys along the same route, using the same protocol, on consecutive days. An example of these replicates over time is given in Figure 4, which displays the dogs per km of street recorded at every survey, both replicates within survey event and repeated surveys over time for the four routes in Panama City.

Regression analysis of the data shown in Figure 4 reveals the Casco Viejo route has shown a statistically significant change in density over time, with a decreasing density of roaming dogs per km of street across the five survey events (*R^2^* = 0.5934, *p* < 0.05); this route covers an area of the city that has undergone intervention over a number of years, including sterilisation of male and female dogs.

**Figure 5:**
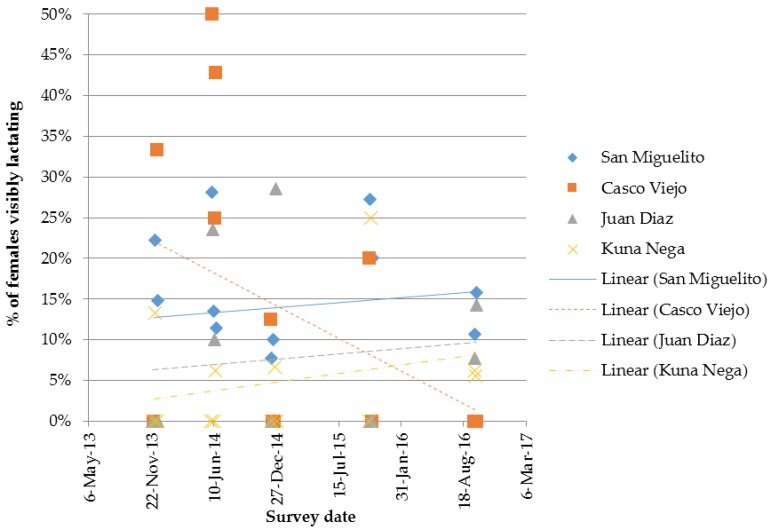
Percentage of females lactating at the time of observation; percentage for each of the four Panama City routes, at each survey event (comprised of two or three replicate surveys per route).

Again, although not statistically significant (*R^2^* = 0.1578, *p* = 0.179), Casco Viejo shows a reduction in percentage of females lactating over time, see Figure 5; percentage lactating is expected to be the first indicator to respond to an intervention that involves spaying of female dogs that roam.

## 4. Discussion

The method of direct observation of roaming dogs along routes provides data for measuring a range of important indicators for roaming dog populations. Its uptake in several locations, including persistent use for monitoring over time, is evidence for its simplicity of implementation.

Where a consistent survey protocol is used to compare changes in the density of dogs observed along standard routes, this represents a novel indicator of the probability of encountering a dog along these routes and their average welfare and reproductive state. As such there is no comparable method against which to validate this method. However, where the method is extended by the use of representative routes and total street length to estimate the total number of dogs visibly roaming in an area, there is the potential to compare this against other approaches. Conducting exhaustive searches of all streets could be used to validate whether the route selected was indeed representative of the true average density across the area. Another potential comparison could be made with the estimate resulting from applying mark-recapture methods [7,8,15], however, as this method suffers from unknown error from potentially violated assumptions, this may not be true validation, but a useful comparison nonetheless.

Implementing this method requires consistent motivation and interest in the data it produces. We have found that those locations that have maintained ongoing monitoring are those that benefit from a local team of surveyors, as opposed to those that require outside personnel. In addition, these local teams require ongoing support in data analysis and interpretation to ensure they are able to use the data in meaningful ways.

A potential weakness of the method is its reliance on consistency in protocol when conducting the street survey to reduce the impact of confounding variables. Hence it is important to ensure surveyors are required to survey at the same time of day and avoid days of unusual human activity, such as national holidays or market days, when dog visibility could be affected. The track taken by the surveyors and their speed is recorded by the OSMtracker app, so that their adherence to the route can be checked and their speed indicates search effort, which needs to be kept constant. To minimise observer differences, local teams are trained by experts in the survey method and, as far as possible, the same observers are used for both the replicate and repeat surveys. Roaming dogs have been shown to display quite prominent seasonal breeding in some locations [16], leading to changes in percentage lactating and density across the year. Hence surveys should be conducted at the same time of year, and where multiple surveys are conducted within the year, comparisons of changes over time must be made between surveys at the same time of year and not across seasons.

The density of roaming dogs, as indicated by the number of roaming dogs recorded per km of street surveyed is sufficiently sensitive to expose significant difference between locations and, in one location, a significant reduction over time. We argue it is also an important and relevant indicator, as it reflects the experience citizens have of roaming dogs in their community, for example as they commute to work and school.

The percentage of females that are visibly lactating has potential as an early stage indicator of change in response to DPM interventions that use spaying of females. If one assumes that the puppies born to roaming females (both owned dogs that are roaming away from home or unowned females) are more likely to be unwanted and suffer high mortality, roaming females become a principle target for spaying. Following implementation of a project that spays female dogs, an observed reduction in lactating females indicates that the roaming dog population is less reproductively active. Reduction in density may follow as roaming dogs die and are not replaced by pups at the same rate due to reduced breeding. However, there may be little reduction in adult roaming dog density if very few pups born on the streets survive and the roaming population is actually sustained by dispersal or abandonment of owned and currently confined dogs. There may even be an initial increase in adult roaming density if female survival improves as a result of spaying.

The importance of the proportion of lactating females in the roaming dog population has also been highlighted by Reece et al. [17]; human animal-bite incidents reported by the SMS Hospital in Jaipur appear to show seasonal variation, with a peak in bites following 10 weeks after the estimated peak whelping time for street dogs in that city. It is hypothesised by the authors that maternal defensive aggression may be the motivation for at least some of these bites from dogs, as this peak occurs when there will be the highest number of puppies at 2–3 months, an age when they are visible and attractive to people, yet still under protection of their dam.

The indicators of body condition score and skin condition are basic measures of welfare state; there was a significant difference in the percentage of dogs with a visible skin problem between locations. Many DPM interventions aim to improve welfare and hence these indicators may provide valuable evidence of impact where they show a change over time. Dogs with an emaciated body condition (BCS 1) were rare in the surveys presented here, with a maximum of 5.1% recorded on one of the routes in Panama City; most routes did not record any emaciated dogs at all, possibly because a dog in an emaciated state is unlikely to survive for long. Nevertheless, the percentage of emaciated dogs is a valuable indicator as this body condition suggests a serious welfare problem that an intervention may be aiming to address. However, where this percentage is very low, the combined percentage of dogs that are emaciated or thin (BCS 2) can also be monitored. Although a thin dog may not necessarily be suffering a serious welfare problem, a high percentage of dogs that are thin or emaciated suggests the population is challenged in terms of health or access to resources. In some locations, such as Kathmandu, where observers are recording high numbers of dogs during the survey, the team may elect to only record the presence of emaciated dogs, because of the time required to assess and record dogs with BCS 2–5.

A limitation of this method is that it does not address the issue of the composition of the roaming dog population with regards to ownership status. As described in the introduction, it is known that a proportion of owned dogs will roam on public streets without owner supervision; this proportion varies considerably between locations and will depend on attitudes of both owners towards confinement and neighbours towards roaming dogs, as well as the structure of homes and fencing which will have cultural and economic drivers. Estimating the ratio of owned to ownerless dogs within a roaming dog population is hypothetically possible by utilising methods of mark-resight combined with a questionnaire of owners; where a known number of the owned dog population that is reportedly allowed to roam is marked, followed by street surveying where observers count roaming dogs and record whether they are marked or unmarked. However, this method is subject to several assumptions and requires intensive effort to implement a questionnaire, mark dogs and survey streets; hence it was considered beyond the scope of a resource efficient method for measuring change over time. Arguably, changes in indicators of dog density, composition and welfare measured through street surveying alone, following the course of an intervention, will provide signs of dog ownership. For example, an intervention that focuses on sterilisation of owned dogs may observe a steep decline in the percentage of roaming females that are lactating, suggesting that the majority of the roaming dogs are indeed owned dogs allowed to roam unsupervised.

## 5. Conclusions

By increasing the use of direct observation of dog density and composition during street counts, the authors hope to contribute to the use of objective data in evaluating and managing DPM interventions. Although the data gathered through this method can be used to compare locations, its real strength is in monitoring changes over time. Since the method is simple and efficient, the surveys can be repeated consistently and relatively frequently along the same routes. Such monitoring has been conducted in several locations; the example of Panama City is provided. However, in most locations only monitoring baselines have been established, because the interventions are in early stages and changes in the roaming dog population are not yet expected.

This method alone will not be sufficient for reliable evaluation or predictions of how a roaming dog population will respond to an intervention; dogs exist within a close relationship with people, those that own, care or dislike them, all impact on their survival, reproduction and welfare. Therefore, additional methods that attempt to measure the attitudes and behaviours of people and how they change in response to intervention will be required for more meaningful evaluation of the mechanisms behind any changes in dog density.

## Figures and Tables

**Table 1:** Indicators relating to density, reproductive activity and welfare of roaming dogs for two or more routes in seven locations; resulting from application of the method of direct observation of dogs along public streets. BCS, body condition score.

Country	Route Type	Name of Route	Date Surveyed	Dogs Per km	% Lactating	% BCS 1	% BCS 1 or 2	% Visible Skin Condition
Bosnia	Hotspot	Kljuc	16 June	9.07	0.0%	0.0%	0.0%	4.2%
		Mrkonjic Grad	16 June	0.78	0.0%	0.0%	0.0%	
		Trebinje	16 September	1.49	11.1%	0.0%	17.9%	10.7%
Bogatic municipality, Serbia	Representative	Badovinci	16 May	0.855	50.0%	0.0%	4.2%	0.0%
		Bogatic		0.6	33.3%	0.0%	11.1%	0.0%
		Crna Bara		0.52	0.0%	0.0%	0.0%	0.0%
		Dublje		0.145	0.0%	0.0%	5.6%	0.0%
		Klenje		0.72	0.0%	0.0%	18.8%	0.0%
Constanta county, Romania	Hotspot	Cernavoda	16 July	4.79	6.8%	0.0%	8.3%	1.4%
	Representative	Agigea	16 September	6.52	25.9%	0.0%	44.4%	18.5%
Panama City, Panama	Representative	San Miguelito	November–December 2013	5.62	11.1%	4.0%	38.7%	28.2%
		Casco Viejo		1.07	8.0%	2.3%	15.9%	11.8%
		Juan Diaz		1.87	0.0%	5.1%	22.0%	22.8%
		Kuna Nega		5.83	4.6%	2.5%	18.6%	14.5%
Puerto Rico	Representative	Aguadilla	14 March	1.13	0.0%	0.0%	2.6%	9.4%
		Fajardo/Ceiba		1.73	4.3%	0.0%	11.3%	22.7%
		Toa Alta		1.73	0.0%	0.0%	2.0%	9.5%
San Jose, Costa Rica	Representative	San Jose	14 June	2.59	8.8%	0.0%	5.6%	6.2%
		Heredia Y Belen		1.50	0.0%	0.0%	2.0%	6.3%
		Cartago		3.09	11.1%	1.0%	9.0%	5.1%
		Alajuela		2.28	20.0%	1.1%	9.6%	6.2%
		Rancho Redondo		4.09	6.5%	1.3%	6.4%	2.0%
Kathmandu, Nepal	Representative	Zone 1	16 March	12.30	12.3%	0.0%	*	0.9%
		Zone 2		27.14	6.2%	0.1%		3.3%
		Zone 3		12.28	6.0%	0.0%		2.7%
		Zone 4		14.41	6.6%	0.1%		2.6%
		Zone 5		15.66	4.1%	0.1%		3.0%
		Zone 6		8.40	8.3%	0.0%		6.5%
		Zone 7		11.37	20.3%	0.0%		1.8%
		Zone 8		11.76	10.3%	0.0%		0.9%

* Only emaciated dogs with BCS 1 had their body condition score recorded during the survey in Kathmandu; this was due to the high number of roaming dogs to record within limited survey time.

**Table 2:** Monitoring data resulting from repeat surveying along standard routes in five locations.

Country	Route Type	Name of Route	13 December	14 June	14 December	15 October	16 September
Panama City, Panama	Representative	San Miguelito	5.62	5.453	5.887	5.73	5.895
		Casco Viejo	1.07	1.03	0.95	0.79	0.44
		Juan Diaz	1.87	2.99	2.735	2.035	1.805
		Kuna Nega	5.83	4.8167	3.883	4.73	4.435
Country	Route Type	Name of Route	14 March	15 April	16 March		
Puerto Rico	Representative	Aguadilla	1.13	0.98	0.60		
		Fajardo/Ceiba	1.73	1.23	1.25		
		Toa Alta	1.73	1.20	1.04		
Country	Route Type	Name of Route	16 June	16 September			
Bosnia	Hotspot	Kljuc	9.07	7.04			
Country	Route Type	Name of Route	16 July	16 October			
Constanta county, Romania	Hotspot	Cernavoda	4.79	4.34			
Country	Route Type	Name of Route	16 March	16 November			
Kathmandu, Nepal	Representative	Zone 1	12.30	15.45			
		Zone 2	27.14	29.565			
		Zone 3	12.28	12.675			
		Zone 4	14.41	13.54			
		Zone 5	15.66	18.42			
		Zone 6	8.40	10.905			
		Zone 7	11.37	14.425			
		Zone 8	11.76	12.835			
